# Dereplication-assisted culturomics enables strain-level ecological analysis of the human gut microbiome

**DOI:** 10.1080/19490976.2026.2681840

**Published:** 2026-05-28

**Authors:** Soyoung Yeo, Hyunjoon Park

**Affiliations:** a Department of Agricultural Biotechnology, College of Agriculture and Life Sciences, Seoul National University, Seoul, South Korea; b Research Institute of Eco-friendly Livestock Science, Institute of Green-Bio Science and Technology, Seoul National University, Pyeongchang, Gangwon-do, South Korea

**Keywords:** Human gut microbiome, culturomics, MALDI-TOF MS dereplication, operational isolation unit, strain-level ecology

## Abstract

Recent advances in culturomics have enabled large-scale recovery of microbial isolates from the human gut, generating extensive culture collections that bridge metagenomic predictions and experimental validation. However, these isolate resources remain largely underutilized, as conventional culturomics prioritizes the discovery of novel species while massive collections of commensal isolates persist as unexplored biological datasets. Dereplication, particularly based on MALDI-TOF MS spectral features, has been largely regarded as a logistical tool for managing redundancy rather than an analytical asset. Here, we reposition dereplication as an analytical framework for interpreting large-scale culturomics datasets and resolving strain-level ecological patterns. We applied the SPeDE pipeline to a comprehensive collection of 2,231 isolates, including *Bifidobacterium* spp. and *Enterococcus faecium*, recovered from healthy donor feces. Spectrum-derived operational isolation units (OIUs) revealed host-associated strain-level repertoires and lineage-like clustering within species. Notably, distinct spectral clusters observed in *E. faecium* corresponded to clade-level patterns identified through shotgun metagenomic analysis. These findings demonstrate that dereplication-assisted culturomics can extend beyond redundancy control to enable high-resolution ecological interpretation of cultured microbiome datasets. By reframing dereplication as a bridge between large-scale isolate generation and strain-level microbiome ecology, this study outlines a conceptual and practical direction for the next phase of human microbiome research in the post-culturomics era.

## Introduction

The human gut microbiome plays a crucial role in host physiology, metabolic regulation, and immune homeostasis.^1^ Although recent advances in sequencing technologies have significantly improved our understanding of microbial community composition, most analyzes still focus primarily on the species level.^2^ This species-centric perspective inherently assumes homogeneity within taxa; however, this assumption can overlook functional variation among strains within the same species in host–microbe interactions and disease outcomes. The actual functional unit of the microbiome is increasingly recognized as the strain rather than the species, as strain-specific traits critically determine outcomes such as pathogenicity, metabolic capacity, and host interactions.^3^ Therefore, understanding of the microbiome necessitates resolving microbial diversity at the strain level.

High-resolution metagenomic approaches have enhanced *in situ* strain profiling, yet these culture-independent methods remain limited by the incompleteness of assembled genomes and by the challenges of experimental validation.^2^ ^,^ ^3^ The ongoing constraints have renewed interest in integrating culture-dependent approaches with existing metagenomic frameworks. Culturomics is a high-throughput culture technique that uses matrix-assisted laser desorption/ionization time-of-flight mass spectrometry (MALDI-TOF MS) for rapid identification, enabling the large-scale collection of culture strains for microbiome studies.^4^ Nevertheless, it is evident that current culturomics remains a bottleneck to translating large isolate collections into strain-level biological insights. Although automated high-throughput systems and streamlined protocols have mitigated practical inefficiencies,^5^ ^,^ ^6^ this increased capacity has introduced a paradoxical challenge: the massive accumulation of duplicate isolates, which scale directly with total throughput. Strain redundancy increases resource burden and complexity of culture collection management, hindering the efficient selection of representative strains for downstream analysis. Consequently, a robust dereplication framework is required to unlock the biological value embedded in large-scale culturomics datasets.^7^ Several dereplication frameworks based on MALDI-TOF MS spectra have been introduced, including the SPeDE algorithm developed by Vandamme et al.^8^ ^,^ ^9^.

**Figure 1.**
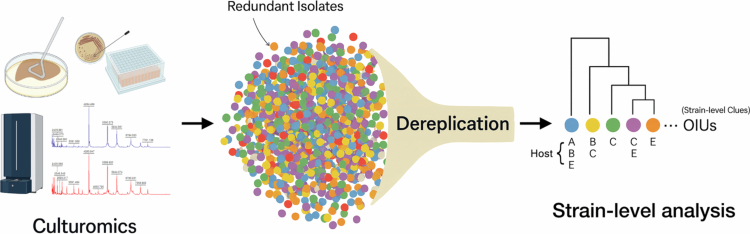
Overview of the analytical framework linking high-throughput culturomics to strain-level ecological pattern analysis via MALDI-TOF MS-based dereplication and operational isolation unit (OIU) generation.

While previous studies have primarily focused on dereplication for the technical management and storage of culture collections, we aim to demonstrate its potential as a powerful analytical lens for uncovering host-associated strain-level patterns and for understanding the fine-scale ecological structure of the human gut microbiome. In this study, we applied an operational isolation unit (OIU)-based dereplication framework to a high-density subset of 2,231 isolates selected from our library of over 22,000 human gut isolates.^6^ ^,^ ^10^ By shifting the analytical unit from raw isolates to spectrum-derived OIUs leveraging high-resolution spectral features, we demonstrate that dereplication can transcend its conventional logistical role to unlock the previously unexploited biological value of commensal isolates. This approach provides actionable ecological insights into strain-level microbiome patterns, establishing a robust foundation for precision microbiome research (Figure 1A).

## Materials and methods

### Bacterial isolates and MALDI-TOF MS spectral acquisition

The isolates analyzed in this study were selected from an extensive human gut microbial library previously established by our group.^6^ ^,^ ^10^ The collection comprises a total of 22,236 isolates, including 14,095 isolates from inflammatory bowel disease (IBD) patients and 8,141 isolates from healthy individuals. To apply the OIU-based analytical framework in a well-resolved dataset, we prioritized a high-density subset from the healthy cohort.^6^ We selected donors (*n* = 8) who provided sufficient intra-host representation (at least ten isolates per host), resulting in a comprehensive dataset of 2,231 isolates (*Bifidobacterium* spp., *n* = 1,097; *Enterococcus faecium*, *n* = 1,134). These taxa were selected because of their widespread presence in the human gut, which necessitates analytical approaches capable of capturing structured patterns among isolates from multiple hosts, beyond simple species-level identification.

All isolates were cultured using a standardized culturomics workflow in which culture media (mGAM), incubation at 37 °C under anaerobic conditions, isolation procedures, colony selection, and subculturing steps were applied identically across all samples to ensure comparability of downstream MALDI-TOF MS spectra.^6^

Spectra were acquired using a MALDI-TOF MS system (Bruker Biotyper Sirius, Bruker Daltonics, Bremen, Germany) containing the MBT 8,468 MSPs library. For analysis, a single colony was thinly smeared onto a target plate, overlaid with 1 μl of 70% formic acid, and air-dried at room temperature. Subsequently, 1 μl of matrix solution (*α*-cyano-4-hydroxycinnamic acid, HCCA) prepared in 50% acetonitrile, 2.5% trifluoroacetic acid, and deionized water was added and allowed to dry prior to spectral acquisition.

### MALDI-TOF MS dereplication and clustering

All MALDI-TOF MS spectra were analyzed using the SPeDE pipeline to perform dereplication and spectrum-based clustering, with default parameters (density = 700 ppm, cluster threshold = 75%, local threshold = 50%, and S/N cutoff = 30).^8^ ^,^ ^9^ To ensure high-quality microbial identification and robust clustering, we selected only isolates with MALDI-TOF MS scores ≥ 1.9. Furthermore, to adequately represent intra-host diversity, we included only hosts that contributed at least ten isolates. Following the SPeDE algorithm, unique spectral feature (USF) matrices were constructed to compare isolates at the genus level across the four *Bifidobacterium* species (*B. adolescentis*, *B. bifidum*, *B. longum*, and *B. pseudocatenulatum*) and at the single-species level for *E. faecium*. Clusters comprising indistinguishable spectra were defined as the same OIU, with the spectrum containing the highest number of USFs designated as the OIU reference spectrum (Supplementary Tables 1 and 2). Notably, the resulting USF profiles and OIU assignments served as the basis for hierarchical clustering, network analysis, and downstream diversity assessments at the OIU level. The relationships between the dereplicated OIUs were analyzed using dendrogram and network analysis scripts provided by the developers of SPeDE workflow (https://github.com/LM-UGent/SPeDE).

### Statistical analysis and visualization

All statistical analyzes and visualizations were performed in R (version 4.3.2). For each species, intra- and inter-OIU distances were compared using the Wilcoxon rank-sum test (z score and—log_10_ *P*), and effect sizes were calculated using Cliff’s *δ* statistic. The magnitude of the effect size was interpreted based on established benchmarks, where |δ| ≥ 0.47 was considered to indicate a substantial separation between groups. Dimensionality reduction using *t*-distributed stochastic neighbor embedding (*t*-SNE) was performed to visualize isolate distribution and host-associated clustering patterns.

### Shotgun metagenomic analysis

To support dereplication-based OIU-level structure, shotgun metagenomic data from one donor (HC3) were analyzed. Taxonomic profiling was conducted using MetaPhlAn 4^11^ within the Biobakery WGS pipeline to achieve clade-level resolution, utilizing the Nephele platform (https://nephele.niaid.nih.gov/) with default parameters based on database (mpa_vJun23_CHOCOPhlAnSGB_202307).

## Ethics statement

Human fecal samples from healthy donors used for isolate recovery were collected under a protocol approved by the Institutional Review Board of Soon Chun Hyang University Bucheon Hospital (IRB no. 2021-01-028-002).^6^ The present study analyzed previously collected and anonymized bacterial isolates and did not involve additional human subject recruitment.

## Results

**Figure 2.**
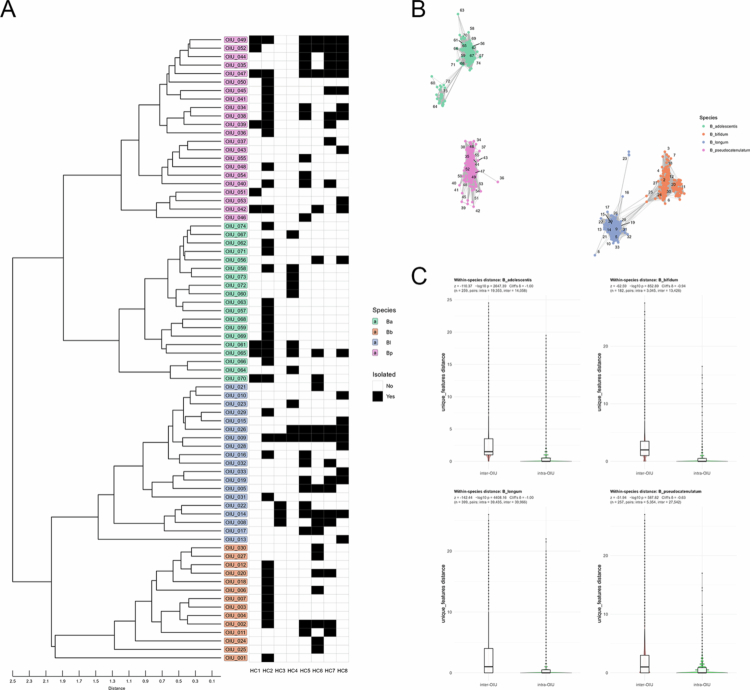
Host-associated strain-level patterns revealed by OIU-based dereplication of *Bifidobacterium* isolates. (A) Distribution of *Bifidobacterium* OIUs across donors. The dendrogram (left) represents spectral distances, while the heatmap (right) indicates the presence (black) or absence (white) of each OIU across hosts (HC1–HC8). Colored bars denote species assignments (Ba: *B. adolescentis*, Bb: *B. bifidum*, Bl: *B. longum*, Bp: *B. pseudocatenulatum*). (B) Spectrum-based network analysis of all *Bifidobacterium* isolates derived from MALDI-TOF MS distances. Nodes are colored by species, and labels indicate OIU numbers. (C) Within-species spectral distance comparisons among *Bifidobacterium* isolates. Violin plots and boxplots compare spectral feature distances between pairs of isolates from different OIUs (inter-OIU, red) versus pairs within the same OIU (intra-OIU, green). Statistical comparisons between intra- and inter-OIU distances were performed as described in Materials and methods.

Dereplication of isolates into OIUs revealed host-associated organization of strain-level patterns. We first performed dereplication on a collection of 1,097 *Bifidobacterium* isolates, comprising four species: *B. adolescentis*, *B. bifidum*, *B. longum*, and *B. pseudocatenulatum*. This analysis categorized the total isolates into 74 distinct OIUs (Supplementary Table 1). The resulting dendrogram demonstrated clear species-level resolution, supporting the reliability of OIU-based dereplication clustering (Figure 2A). A similar pattern was observed in a spectrum-based network analysis (Figure 2B). Regarding the host distribution of OIUs, we observed substantial variations in OIU composition across individual hosts, indicating host-associated variation (Figure 2A). Forty-four OIUs were unique to a single host, while the remaining OIUs were observed in two or more hosts (unique OIU ratio: *B. pseudocatenulatum* = 50.0%, *B. adolescentis* = 73.7%, *B. longum* = 47.4%, *B. bifidum* = 71.4%). Pairwise comparisons of unique feature distances among all isolates revealed that inter-OIU distances were consistently and significantly larger than intra-OIU distances within each species (Figure 2C). These results indicate that spectrum-based OIUs capture host-associated spectral patterns consistent with structured phenotypic differentiation at the strain-level. Importantly, dereplication revealed ecological patterns that are not apparent when isolates are analyzed at the species level alone.

**Figure 3.**
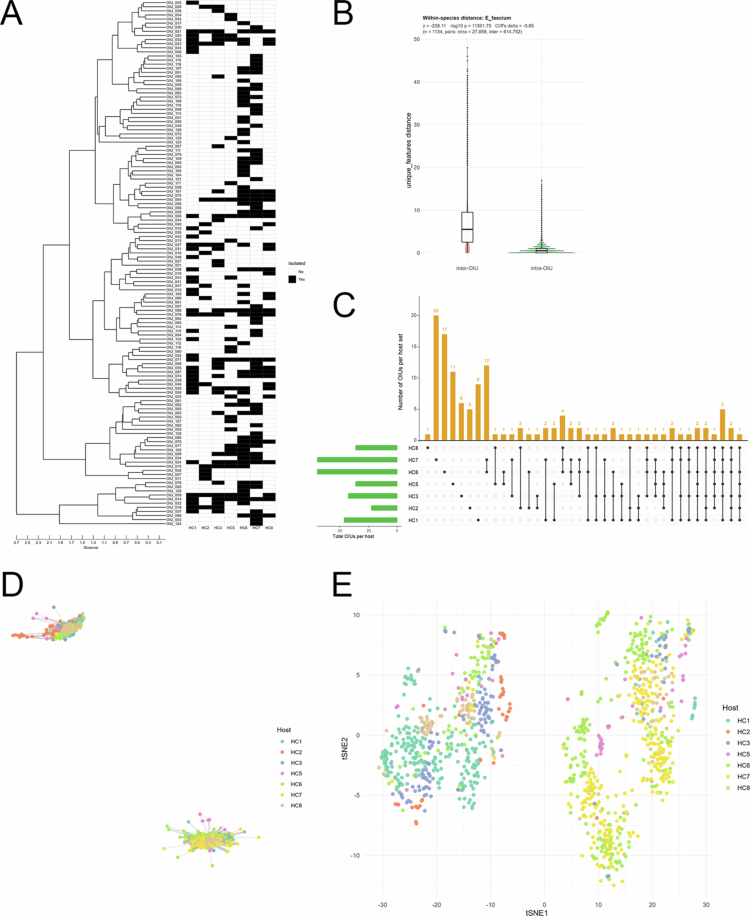
Intra-species ecological patterns of *Enterococcus faecium* across donors revealed by OIU-based dereplication. (A) Distribution of *E. faecium* OIUs. (B) Within-species spectral distance comparison for *E. faecium*. Statistical comparisons between intra- and inter-OIU distances were performed as described in Materials and methods. (C) UpSet plot showing OIU sharing and prevalence across hosts for *E. faecium*. The matrix indicates host combinations sharing specific sets of *E. faecium* OIUs, while the bar charts show the total OIUs per host (left) and the number of OIUs within each intersection set (top). (D) Spectrum-based network analysis based on MALDI-TOF MS distances for all isolates of *E. faecium*, with nodes colored by host. (E) *t*-distributed Stochastic Neighbor Embedding (*t*-SNE) of all *E. faecium* isolates, colored by host to visualize clustering and host-associated distribution patterns.

Next, to investigate whether OIUs capture within-species diversity patterns, we analyzed an extensive collection of *E. faecium* isolates (*n* = 1,134). The 128 identified *E. faecium* OIUs exhibited distinct compositional patterns across hosts (Figure 3A and Supplementary Table 2). Statistical comparisons between intra- and inter-OIU distances showed that inter-OIU distances were significantly greater than intra-OIU distances, supporting the presence of structured intra-species patterns captured by OIU-based clustering (Figure 3B). A total of 81 OIUs were identified as host-restricted, and the number of unique OIUs per host varied significantly (ranging from 1 in HC8 to 20 in HC7). In contrast, a notable portion of OIUs was shared among donors. These findings suggest that while the strain-level patterns are host-associated, they are not entirely exclusive to individual hosts (Figure 3C). These shared OIUs should be interpreted with caution, as they may reflect not only biological overlap but also technical or sampling-related effects. A particularly intriguing finding from the OIU-based spectral similarity network analysis was the partitioning of *E. faecium* isolates into two distinct, cluster-level grouping (Figure 3D). The separation was consistently observed when the same spectral data were visualized using *t*-SNE (Figure 3E). These two clusters maintained independent structures with minimal overlap in spectral peak patterns and OIU composition, suggesting that OIU-based networking can capture intra-species population structure.

**Figure 4.**
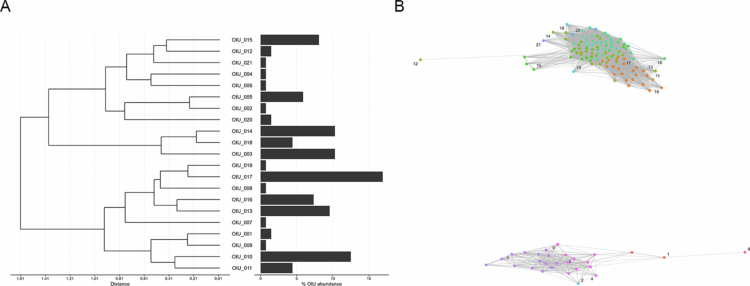
Within-host ecological patterns of *Enterococcus faecium* in donor HC3 revealed by spectral dereplication. (A) Spectral distance relationships among OIUs identified in donor HC3, paired with their relative OIU abundances. The dendrogram illustrates hierarchical clustering of OIUs recovered from this host. (B) Spectrum-based network analysis of all isolates in donor HC3. Nodes are colored by OIU assignment.

Thus, we investigated whether the OIU-based clusters observed in *E. faecium* correspond to genomic clades detectable through metagenomics by conducting an in-depth analysis of a single donor, HC3. The 136 isolates recovered from this host were classified into 21 OIUs (Figure 4A). Both dendrogram and network analyzes based on spectral similarity revealed two clearly separated clusters (Figure 4A and B). To provide a complementary genomic perspective, we performed shotgun metagenomic sequencing on the corresponding fecal sample from HC3. The metagenomic analysis, utilizing MetaPhlAn4 within the Biobakery workflow, identified two major *E. faecium* clades (SGB7968 and SGB7967). Given the properties of the species-level genome bins (SGB) taxonomic system,^12^ although a strict one-to-one correspondence between individual OIU-based clusters and metagenomic clades has not been definitively established, nonetheless, the congruence between the two independent modalities supports the biological plausibility of OIU-based clustering. This result supports the notion that OIU-based clusters can capture biologically meaningful patterns consistent with diversity inferred from metagenomic analyzes.

## Discussion

This study demonstrates that MALDI-TOF MS–based dereplication can serve as an analytical framework for understanding the strain-level ecological patterns of the human gut microbiome. Unlike previous studies that primarily used dereplication to enhance technical efficiency during the isolation of diverse environmental, food, or clinical samples,^8^ ^,^ ^9^ ^,^ ^13^ ^,^ ^14^ we applied this approach to the analysis of a large-scale spectral dataset of human gut culturomics under standardized conditions. Consequently, our findings position dereplication as a critical bridge connecting culturomics with strain-level microbial ecology.

Our dereplication-assisted culturomics approach yields two significant insights into human gut bacterial ecology. First, the distinct host-associated OIU patterns observed in both *Bifidobacterium* spp. and *E. faecium* provide culture-based evidence of structured variation at the strain-level across individual hosts. This finding is consistent with the concept of personalized strain-level patterns, as previously inferred from metagenomic studies. Second, OIU-based network and cluster analysis can identify subtle strain-level patterns within a single species.

This approach is instrumental in advancing to “post-culturomics” experiments and applications by using vast strain resources generated through high-throughput culturing. For instance, it enables the identification of group-specific OIUs even within cohorts that appear indistinguishable based on species-level relative abundances in metagenomic data. These representative OIUs can be prioritized for phenotypic characterization to identify isolates of interest, thereby reducing experimental burden and improving the efficiency of downstream characteristics, which may ultimately support microbiome-based applications. Additionally, this framework may be well-suited to fecal microbiota transplantation studies to monitor donor-associated strain-level dynamics in recipients and to pathobiont research to identify potentially high-risk lineages within a single species.

Despite its advantages, several limitations of this approach should be acknowledged. First, the dereplication workflow is inherently sensitive to bacterial culture conditions, necessitating highly standardized protocols to ensure reproducible spectral patterns.^7^ ^,^ ^9^ Second, the analysis is restricted to the culturable fraction of the microbiota and thus does not capture the full diversity of gut microbes.^15^ In addition, differences in sampling depth, including uneven isolate counts across hosts, may influence the observed distribution of OIUs. Our previous study indicates that, beyond a certain threshold, isolate counts are sufficient to capture representative diversity patterns.^6^ ^,^ ^10^ Third, although the OIU serves as a data-driven phenotypic grouping of isolates, it is important to recognize the inherent conceptual boundary between these units and genomically defined strains. While the OIU provides an effective proxy for capturing structured variation at the strain level, MALDI-TOF MS primarily identifies high-abundance proteins (e.g., ribosomal proteins) and thus may not resolve fine-scale genomic variations that do not manifest in the proteomic phenotype, such as minor single-nucleotide polymorphisms or the presence of mobile genetic elements. This limitation is inherent to the current state of MALDI-TOF MS technology, as evidenced by recent large-scale benchmarking studies demonstrating that while the method is highly reliable for species-level identification, its performance in distinguishing subtle intra-species variations is limited, and spectral similarity does not necessarily preserve established phylogenetic relationships.^14^ Consequently, the OIU framework should be viewed as an imperfect but highly practical and scalable approach for addressing the analytical bottlenecks in large-scale culturomics. In the absence of fully integrated high-throughput and cost-effective genomic approaches, this spectrum-based clustering remains the most viable bridge for identifying consistent, non-random structural patterns within vast isolate collections. Nevertheless, the clear separation observed between intra- and inter-OIU distances in our dataset indicates that spectrum-based clustering captures consistent and non-random structural patterns among isolates, supporting its utility as an intermediate analytical framework for large-scale culturomics (Figures 2C and 3B). Accordingly, MALDI-derived clustering should be interpreted as a functional phenotypic categorization rather than a definitive reflection of comprehensive genomic differentiation. Therefore, follow-up multidimensional assessments are required to further evaluate the genetic and functional characteristics associated with individual OIUs.

In conclusion, this study highlights that MALDI-TOF MS-based dereplication and analysis transcends mere technical streamlining, offering significant analytical value for interpreting the strain-level ecological patterns of the human gut microbiome. By integrating this approach within a culturomics framework, we outline a conceptual and practical foundation that links high-throughput culturing to precision microbiome ecology. This framework paves the way for diverse future microbiome research, in which strain-level interpretation and validation are essential for understanding complex host-microbe interactions.

## Supplementary Material

Supplementary Material Supplementary Data.xlsx

## Data Availability

Shotgun metagenome sequencing data for one donor (HC3) has been submitted to NCBI, SRA accession no. SRR37288113 under BioProject no. PRJNA975692. All other data are contained within the manuscript and supporting information.
